# Supplementary material for: Flight Dispersal in Supratidal Rockpool Beetles
Source: Insects. 2024 Feb 20;15(3):140. doi: 10.3390/insects15030140 (PMC10971659; doi:10.3390/insects15030140)
Supplement: Supplementary file 1 [file insects-15-00140-s001.zip › insects-2853755-supplementary.pdf]

Table S1. Sampled localities (ordered from north to south) and number of genetically analysed individuals of each species by sex.

Locality (from N to S)	Coordinates (LAT, LON)	*O. quadricollis* (♂ / ♀)	*O. lejolisii* (♂ / ♀)
Moraira (Alicante)	38.684856, 0.125222	2 / 2	3 / 2
La Illeta (Alicante)	38.431639, -0.380675	3 / 2	4 / 1
Santa Pola (Alicante)	38.197110, -0.514417	3 / 2	1 / 3
Punta del Cocedor (Murcia)	37.749964, -0.728281	3 / 2	1 / 2
Percheles (Murcia)	37.529250, -1.379486	2 / 3	1 / 0
Cala de las Pulgas (Murcia)	37.469876, -1.470352	4 / 1	3 / 2
Cala Panizo (Almería)	37.318997, -1.700153	4 / 1	3 / 2
Cala de las Conchas (Almería)	37.284133, -1.730989	1 / 3	0 / 1
El Playazo (Almería)	36.862806, -2.003569	5 / 0	0 / 1
Playa Rijana (Granada)	36.709206, -3.392281	4 / 1	1 / 1
Velilla (Granada)	36.744903, -3.661633	5 / 0	0 / 5
Nerja (Málaga)	36.740919, -3.884114	2 / 3	1 / 4
Cala Milla de Plata (Cádiz)	36.306514, -5.260192	2 / 1	0 / 2
Isla de las Palomas (Cádiz)	36.001922, -5.612436	3 / 2	1 / 3

Table S2. Details and sequences of selected loci of O. *lejolissi* and *O. quadricollis* from García-Meseguer et al. (2023b) for Fst calculation.

Locus		Primer sequences 5′- 3′	Repeat type	Size bp	No. of alleles
O. lejolisii					
os_705252	F<br>R	ACAACAATCATGGAGGTCCG<br>CGTAGGTCGAAAACTAATGTCCTC	(AAT)9	243-261	6
os_7126762	F<br>R	ATTACAGTGCGTCTGAGTGC<br>AGACAACTTATTCCAACGAAGC	(AAT)8	85-97	4
os_8667552	F<br>R	CACCGATTGTATCAGCAGCC<br>TGAACAAATAAAGTGCGCTTCTTC	(TAA)8	146-156	4
os_8902151	F<br>R	CACAGGTCGGGGCTAAAATG<br>TCGAAAACTTTAACCCAAGATTGC	(ATA)8	138-144	3
os_10996921	F<br>R	TGCCACTTGCTCGAAGAAAC<br>TCTCGTAAATTTTGTAGAGTTGGGG	(TTA)8	173-186	5
os_12251791	F<br>R	AACAAAAGGCGCTTATGACG<br>AGAACAATTACGTTCTACAATGTGC	(AT)27	125-130	2
O. quadricollis					
**Locus**		**Primer sequences 5′- 3′**	**Repeat type**	**Size bp**	**No. of alleles**
Oq_7434814	F<br>R	CACTCCAATTTGAACTACAATAAGTCC<br>AGCATCCTCTGGTGATGTCC	(TAT)8	232-247	6
Oq_14573024	F<br>R	CTACATCCTGATCGGAGCCC<br>CACCATCCAGAACACCAAGC	(ATA)9	180-194	6
Oq_16109093	F<br>R	CGACCCTCTTCAATACCAAGC<br>GTCCACCAAAGAACGAGGAC	(ATT)9	222-231	4
Oq_20150463	F<br>R	TCCGTTTGAGAGTAGCACCC<br>GGGACGGTATATGGGGATGG	(AGT)10	201-210	4

Oq_21849033	F<br>R	ATGTTTGGACCGCCATTGTG<br>TGTTAGTTTGATGATTTTCTTCGAC	(ATTA)7	118-143	2
Oq_23532364	F<br>R	AACACTCCTAGTGCTCGCTC<br>ATCTGGAGCTCATATCCGCC	(TTA)8	216-225	4
